# Asymmetric Total Synthesis of (+)-21-*epi*-Eburnamonine Via a Photocatalytic Radical Cascade Reaction

**DOI:** 10.1007/s13659-020-00276-8

**Published:** 2020-11-05

**Authors:** Yuan Huang, Fanglin Xue, Hengmao Liu, Fei Xue, Xiao-Yu Liu, Hao Song, Yong Qin

**Affiliations:** Key Laboratory of Drug-Targeting and Drug Delivery System of the Education Ministry and Sichuan Province, and Sichuan Research Center for Drug Precision Industrial Technology, West China School of Pharmacy, Sichuan University, Chengdu, 610041 Sichuan China

**Keywords:** Eburnamine-vincamine alkaloids, Photochemistry, Radical cascade reaction, Johnson-claisen rearrangement

## Abstract

An asymmetric total synthesis of (+)-21-*epi*-eburnamonine has been achieved. Key features of the synthesis include a visible-light photocatalytic intra-/intramolecular radical cascade reaction to assemble the tetracyclic ABCD ring system, and a highly diastereoselective Johnson-Claisen rearrangement to establish the C20 all-carbon quaternary stereocenter.

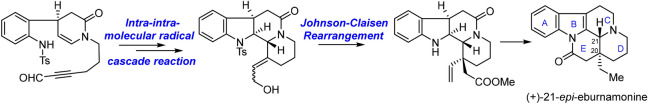

## Introduction

**Fig. 1:**
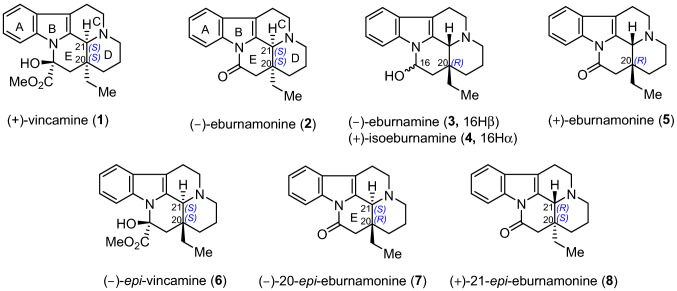
Structures of representative Eburnamine-Vincamine alkaloids and non-natural analogues

The eburnamine-vincamine indole alkaloids are a large group of natural products occurring in the plant family *Apocynaceae* (For selected reviews, see: [1–8]). Featuring the fused-pentacyclic skeleton with multiple continuous stereocenters, this compound class has played an important role in natural product chemistry owing to their diverse structures (For selected reviews, see: [1–8]) and medical importance in cell multiplication, cardiovascular system, and brain functions [9, 10]. During the past few decades, the continuing interests in this family regarding either the synthesis or the pharmacological activities have led to abundant research results. The prominent alkaloids (+)-vincamine (Fig. 1, **1**), (−)-eburnamonine (**2**) and their *cis* D/E ring fusion congeners (Fig. 1, **3-5**) exhibit significant cerebral/peripheral vasorelaxation and antihypertensive bioactivities, especially **1** and **2** have been used in the treatment of hypertension [9–12], making them among the most-studied synthetic targets in eburanamine-vincamine family (For selected examples of racemic total synthesis of **1**, see: [13–20]; For selected examples of asymmetric total synthesis of **1**, see: [21–32]; For selected examples of racemic total synthesis of **2**, see: [33–53]; For selected examples of asymmetric total synthesis of **2**, see: [54–62]). Quite a few synthetic non-natural analogues also exert bioactivities; for instance, (−)-20-*epi*-vincamine (**6**) and (−)-20-*epi*-eburnamonine (**7**) with *trans* D/E ring fusion show better peripheral vasodilation than (+)-vincamine (**1**) and (−)-eburnamonine (**2**) [63], and (−)-21-*epi*-vincamine (**8**) displays higher binding affinity to human serum albumin than **2** [63]. However, compared to the *cis* series (For selected examples of asymmetric total synthesis of **1**, see: [21–32]; For selected examples of asymmetric total synthesis of **2**, see: [54–62]), the asymmetric synthesis of the above molecules (i.e., **6**–**8**) with *trans* D/E ring fusion remains limited [63–67]. Among the numerous strategies developed to access eburanamine-vincamine alkaloids, stereoselective construction of the C20 and C21 stereocenters has always been the most difficult point. Particularly, the establishment of the C20/C21 *trans* relative stereochemistry is challenging [67].

**Scheme 1:**
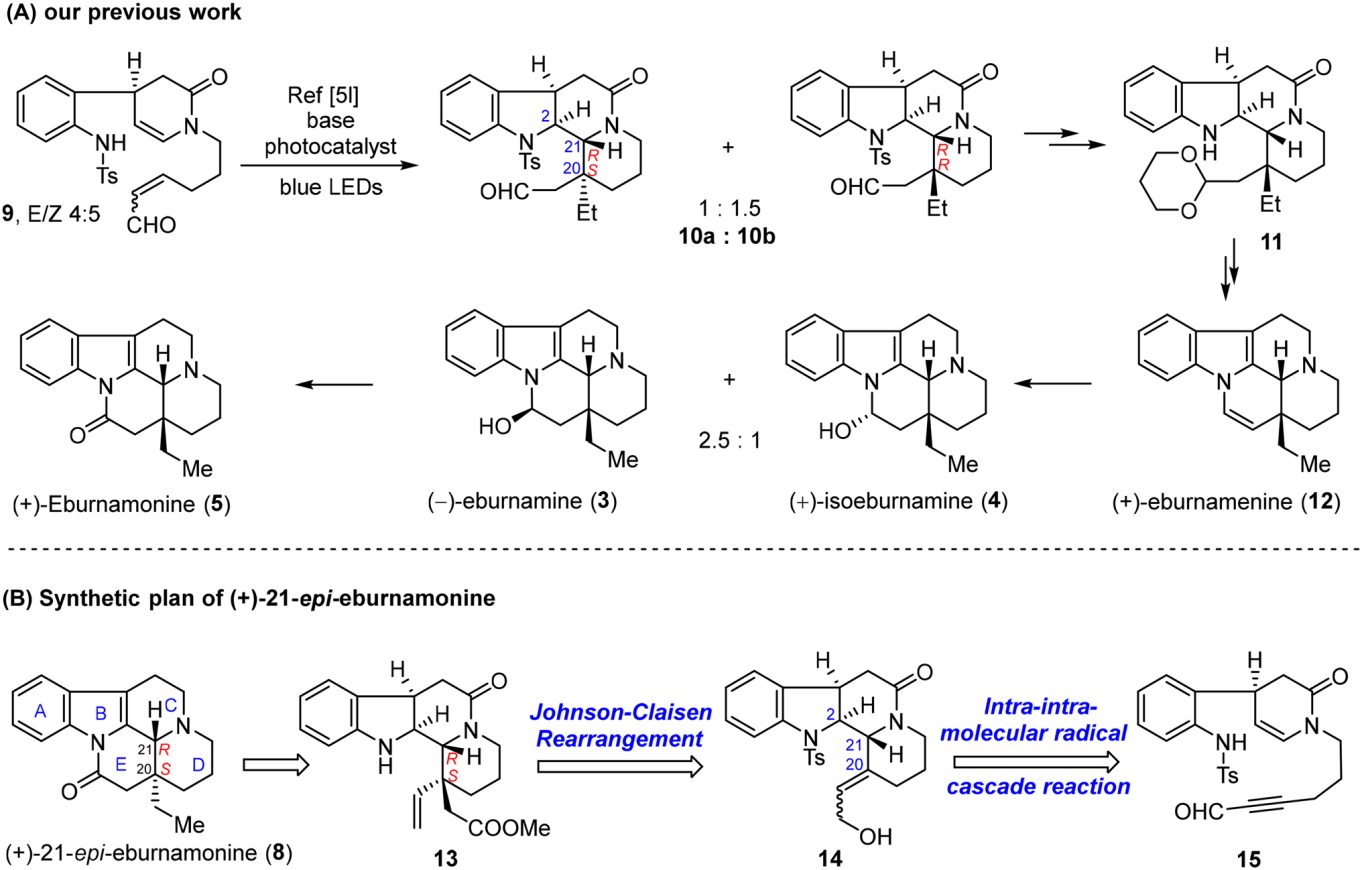
**a** Synthesis of Eburnamonine-vacamine alkaloids via intra-/intramolecular photocatalytic radical cascade reaction developed by our group; **b** Synthetic plan of (+)-21-*epi*-eburnamonine

Attracted by the fascinating structures and prominent biological activities of eburnamine-vincamine alkaloids, our group has devoted to the development of efficient strategies towards natural products belonging to this category [32, 68]. In 2017, we reported three types of nitrogen-centered photocatalytic radical cascade reactions. This unified methodology enabled collective synthesis of 33 monoterpenoid indole alkaloids belonging to four families [32]. Among them, as outlined in Scheme 1a, the intra-/intramolecular cascade reaction of propenal **9** under the irradiation of blue LEDs led to the formation of **10** as a pair of separable diastereomers (d.r. = 1:1.5 at C20), of which the A/B/C/D ring of the pentacyclic eburnane skeleton was assembled in one-pot, with the C21(*R*) stereochemistry established in full control [32]. Tetracycle **10b** with C20 (*R*) stereocenter was further elaborated into a series of eburnamine-vincamine family alkaloids, including (+)-eburanamenine (**12**), (+)-isoeburnamine (**4**), (−)-eburnamine (**3**) and (+)-eburnamonine (**5**). However, the above-mentioned cascade reaction of **9** suffered from unsatisfactory stereocontrol at C20 (d.r. = 1:1.5), which prompted us to seek new synthetic approaches to eburnamine-vincamine alkaloids in a highly stereoselective fashion. Herein, we report our efforts in this regard that led to a stereospecific total synthesis of (+)-*epi*-eburnamonine (20*S*, 21*R*) (**8**).

## Results, Discussion and Conclusion

Outlined in Scheme 1b is our synthetic plan of (+)-21-*epi*-eburnamonine (**8**). We envisioned that the known compound propynal **15** [32] would be an appropriate precursor for construction the A/B/C/D framework of eburnane skeleton through intra-intramolecular photocatalytic radical cascade reaction. The resultant tetracyclic product **14** bears a propenol functionality at C20, which allows us to forge the C20 all-carbon quaternary stereocenter of **13** via Johnson-Claisen rearrangement [62, 69–72]. We supposed that the substrate-controlled stereoselectivity in the rearrangement process could guarantee the desired C20 (*S*) configuration. Furthermore, **13** could be readily transformed into the target (+)-21-*epi*-eburnamonine via intramolecular amidation [67] and subsequent reduction of the vinyl group.

**Scheme 2:**
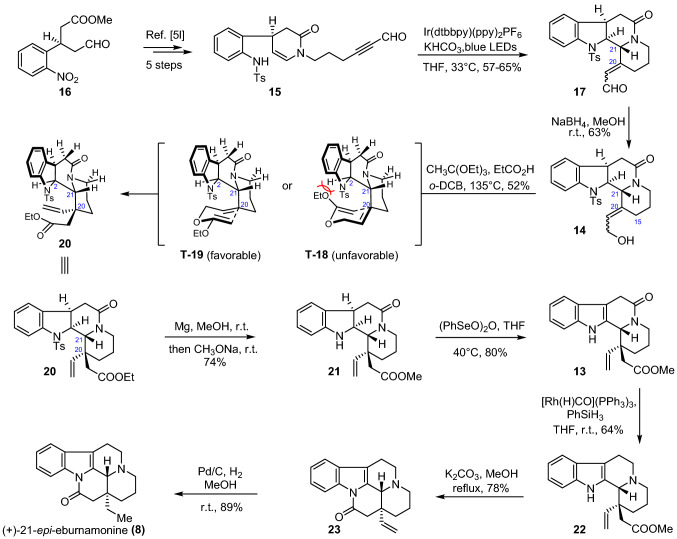
Total synthesis of (+)-21-*epi*-eburnamonine (**8**)

Our synthesis began with preparation of the radical precursor **15** for the devised intra-intramolecular photocatalytic cascade reaction (Scheme 2). Following our previously reported protocols [32], amide **15** was readily obtained from aldehyde ester **16** over five steps on a decagram scale. Upon exposure to the conditions of Ir(dtbbpy)(ppy)_2_PF_6_/KHCO_3_/THF with blue LED irradiation, the intra-intramolecular photocatalytic cascade reaction of **15** proceeded smoothly, delivering a pair of inseparable 2:1 mixtures of *E/Z* isomers **17** in 57–65% yield. It is noteworthy that the above conversion allowed the assembly of the A/B/C/D ring system of eburnane-type scaffold, with stereoselectively construction of C21(*R*) stereochemistry. Subsequently, reduction of propynal **17** with NaBH_4_ afforded the corresponding allylic alcohol **14** as a pair of inseparable geometrical isomers in 63% yield. With **14** available, the vital Johnson-Claisen rearrangement was investigated. Initially, **14** was subjected to CH_3_C(OMe)_3_/EtCOOH/1,2-dichlorobenzene at 135 °C. However, the reaction gave a complex mixture, and the desired product was not observed. Attempts to improve the reaction by screening different acids, solvents or temperatures were also unsuccessful. Fortunately, after changing the trialkyl orthoacetate from CH_3_CH(OMe)_3_ to CH_3_CH(OEt)_3_, the expected rearrangement took place in the presence of EtCOOH in 1,2-dichlorobenzene at 135 °C to give ethyl ester **20** in 52% yield as a single isomer, thereby constructing the C20 all-carbon quaternary stereocenter. Due to the fact that the inseparable mixture of **14** was completely consumed and only one rearrangement product was observed, we supposed that both geometrical isomers of **14** yielded the same compound **20**. The high stereoselectivity of the above-mentioned reaction could be rationalized by the proposed transition-state **T-18** and **T-19**. Presumably, the carbon–carbon bond formation of the rearrangement via **T-18** was hampered by the severe steric repulsion between the ethoxy group and the hydrogen at C2 for both geometrical isomers of **14**, thus the reaction would preferentially occur through transition state **T-19**, favoring the formation of **20** [12].

Next, we turned our attention to the last stage of the synthesis of (+)-21-*epi*-eburnamonine (**9**). To this end, conversion of indoline **20** to indole **13** through a deprotection/oxidation sequence was implemented first. Specifically, upon treatment of **20** with Mg/MeOH followed by addition of CH_3_ONa, the *N*-Ts group was removed along with the transesterification of the methyl ester group, delivering **21** in 74% yield. Subsequent oxidation of indoline **21** with (PhSeO)_2_O led to the formation of indole **13** in 80% yield. Furthermore, by employing the reductive conditions of [Rh(H)CO](PPh_3_)_3_/PhSiH_3_, amide **13** was smoothly converted into amine **22**. Finally, cyclization of the E ring was realized by subjecting **22** to K_2_CO_3_/MeOH under reflux. The delivered pentacycle **23** was then subjected to catalytic hydrogenation in which the vinyl group was reduced to afford the target molecule in 89% yield. Notably, the NMR data of **8** had identical NMR data to (−)-20-*epi*-eburnamonine reported in the literature [66] but opposite optical rotation, which again confirmed the stereochemistry of C20 established in the Johnson-Claisen rearrangement.

In summary, we have disclosed an efficient approach to the asymmetric total synthesis of (+)-21-*epi*-eburnamonine with *trans* D/E ring fusion. From a strategical perspective, the synthesis features a photocatalytic intra-intramolecular radical cascade reaction to assemble the A/B/C/D ring system and a highly diastereoselecive Johnson-Claisen rearrangement to forge the C20 all-carbon quaternary stereocenter. This synthetic strategy provided alternative access towards more derivatives of eburnamine-vincamine alkaloids.
